# In Silico Research Is Rewriting the Rules of Drug Development: Is It the End of Human Trials?

**DOI:** 10.7759/cureus.84007

**Published:** 2025-05-13

**Authors:** Shaheen E Lakhan

**Affiliations:** 1 Medical Office, Click Therapeutics, Inc., New York, USA; 2 Department of Neurology, Western University of Health Sciences, Pomona, USA; 3 Department of Neurology, A.T. Still University School of Osteopathic Medicine in Arizona, Mesa, USA; 4 Department of Neurology, Morehouse School of Medicine, Atlanta, USA; 5 Department of Bioscience, Global Neuroscience Initiative Foundation, Miami, USA

**Keywords:** artificial intelligence, computational biology, digital therapeutics, digital twins, drug development, ethical research, in silico trials, model-informed drug development, personalized medicine, regulatory science

## Abstract

Recent advances in computational biology, artificial intelligence (AI), and regulatory science are rapidly displacing the traditional reliance on animal and early-phase human trials in drug development. In April 2025, the U.S. Food and Drug Administration announced a landmark decision to phase out mandatory animal testing for many drug types, signaling a paradigm shift toward in silico methodologies. This editorial explores how computer-based models, especially digital twins, are emerging as the fourth pillar of biomedical science, capable of simulating complex human systems with remarkable accuracy. Drawing on real-world examples from oncology, neurology, and regulatory submissions, the editorial argues that in silico tools are no longer ancillary but central to modern research. These technologies enable rapid, ethical, and cost-effective drug discovery while offering personalized therapeutic insights. As machine learning, multi-omics integration, and predictive simulations mature, their regulatory credibility grows, particularly in initiatives such as model-informed drug development and digital therapeutics. The editorial concludes by urging the adoption of standardized frameworks, explainable AI, and infrastructure investment to ensure in silico research fulfills its transformative potential. Within a decade, failure to employ these methods may no longer be merely outdated, it may be indefensible.

## Editorial

Modern medicine is accelerating into a digital future, but drug development remains anchored to mid-20th-century paradigms. In an era of personalized, precision care, we still rely heavily on resource-intensive, time-consuming, and ethically fraught human and animal trials. But this is changing. In April 2025, the U.S. Food and Drug Administration (FDA) issued a historic ruling phasing out the requirement for animal testing in many drug development programs, officially marking the beginning of the end for a model that has long been overdue for disruption [1]. This decision was driven by a combination of scientific, ethical, and policy factors, including the limited translational value of animal models, growing evidence in favor of human-relevant systems such as organoids and organ-on-chip technologies, and bipartisan legislative momentum embodied in the FDA Modernization Act 2.0.

Traditional drug development is broken. It takes more than a decade and $314 million to $4.46 billion to bring a single new drug to market [2]. The majority of drugs fail in Phase II or III [3], often due to issues that could have been foreseen with better modeling. This is especially evident in neurodegenerative diseases such as Alzheimer’s, where dozens of drugs, including high-profile candidates such as solanezumab [4] and bapineuzumab [5], have failed late-stage trials despite promising Phase II data. These failures reflect the limitations of existing models to predict long-term cognitive outcomes or stratify patients appropriately. In silico disease progression models and virtual patient simulations could have identified ineffective targets earlier, saving years of time, billions in investment, and the hopes of millions.

Digital twins and the power of simulation

In silico research, the use of computer models to simulate biological systems, is fast emerging as the fourth pillar of biomedical research, alongside in vivo, in vitro, and ex vivo methodologies [6]. These models can emulate organ-level physiology, predict pharmacokinetics, and simulate disease progression across synthetic populations. For example, models have been used to predict outcomes of atrial fibrillation ablation therapy, localize atrial flutter circuits, and augment ECG datasets for training artificial intelligence (AI) in rare cardiac conditions [6]. In addition to modeling disease, in silico platforms such as DeepTox, ProTox-3.0, and ADMETlab are widely used to predict drug toxicity, absorption, distribution, metabolism, excretion, and potential off-target effects, offering scalable alternatives to animal-based toxicology studies.

Once a niche tool, in silico technologies are now maturing into credible, scalable alternatives to early-phase human and animal testing. The FDA’s support for these methods is not just symbolic. Its 2023 guidance on Prescription Drug Use-Related Software (PDURS) [7], the Modernization Act 2.0 [8], and its April 2025 decision to phase out animal testing requirements [9] are clear signals: regulatory science is evolving. Similar efforts are underway at other agencies around the world, including the European Medicines Agency [10] and the Pharmaceuticals and Medical Devices Agency [11], underscoring a coordinated push toward computational evidence as an acceptable foundation for regulatory decision-making.

One of the most promising developments is the rise of digital twins, virtual models of individual patients that integrate multi-omics, including genomics, transcriptomics, and proteomics, biomarkers, lifestyle factors, and real-world data to simulate disease progression and therapeutic response [12]. In fields such as oncology and neurology, digital twins have already predicted outcomes with accuracy rivaling traditional trials. For example, in oncology, researchers have created digital twins of patients’ tumors and their microenvironment to simulate tumor growth and response to immunotherapy, enabling more personalized cancer treatment strategies and aiding in the design of adaptive clinical trials [13]. In neurology, digital twin models have replicated multiple sclerosis progression across diverse patient profiles, allowing prediction of treatment response to disease-modifying therapies and informing real-time adjustments in care pathways [14]. The implications are staggering: a future where each patient receives a treatment simulated and optimized just for them, before they ever take the first pill. Nowhere is this more urgent than in Alzheimer’s disease. Over the past two decades, major pharmaceutical companies have invested billions into amyloid-targeting drugs, only to watch them collapse in late-stage trials [4,5]. In silico models that accurately simulate long-term disease progression and stratify patients by digital biomarkers could have redirected these programs or identified futility earlier [15]. As the field pivots to tau and neuroinflammation targets, simulation offers a lifeline: a chance to get it right before subjecting humans to ineffective or harmful therapies.

With the advent of machine learning and the integration of multi-omics data, genomics, proteomics, and transcriptomics, we can now simulate human biology with astonishing granularity [16]. Models such as AlphaFold have cracked protein folding [17]. Systems biology simulations now replicate complex inter-organ communication. Unlike traditional trials that test one dose in one population, in silico simulations can test thousands of permutations, dosing, timing, comorbidity impact, and polypharmacy interactions instantly. Drug rescue, combination therapy, and rare disease research stand to gain the most from this acceleration.

Toward a new regulatory and ethical standard

The ethical imperative is equally compelling. It is no longer defensible to expose humans or animals to experimental risk when validated in silico alternatives exist. Every unnecessary human or animal trial is a moral failure. The FDA’s animal testing rollback aligns with public sentiment and scientific capability. The time has come to regard computational simulation not as optional, but obligatory. In silico modeling has moved beyond its role as a supportive tool. It is now being used to support label expansions, trial design, and even serve as the primary basis for regulatory submissions. Increasingly, modeling and simulation are being used not just to inform trial design or dosing strategy, but to substantiate safety and efficacy claims directly in lieu of traditional early-phase trials. The FDA has already accepted in silico data as primary evidence in select cases, such as model-informed drug development programs and virtual bioequivalence studies [18]. This marks a pivotal shift: software-derived evidence is no longer supplemental; it is central. The move sets a precedent for future submissions where digital simulations, mechanistic models, and virtual patient cohorts could serve as the core of regulatory dossiers, particularly for rare diseases or precision therapies where conventional trial designs are impractical or unethical.

Prescription digital therapeutics and smart medicines

Prescription digital therapeutics (PDTs) and combination therapies that pair pharmacological agents with software-based interventions (smart medicines) stand to benefit profoundly from the integration of in silico methodologies [19]. These advanced computational tools can be embedded throughout the development lifecycle, from early-stage hypothesis generation and mechanism-of-action modeling to real-time optimization of treatment protocols based on digital phenotyping. By leveraging disease progression models, digital twins, and synthetic patient populations, PDTs can simulate therapeutic effects across diverse clinical scenarios, allowing developers to refine algorithms, tailor interventions to specific subgroups, and identify biomarkers of response before ever enrolling a patient. This level of precision not only bolsters the scientific credibility of these interventions but also generates robust, model-based evidence that can be submitted to regulatory authorities to support safety and efficacy claims.

Moreover, hybrid development strategies that blend real-world clinical data with validated in silico simulations offer a powerful way to accelerate timelines, reduce reliance on large-scale randomized trials, and minimize development costs. For regulatory submissions, these approaches can substantiate dose optimization, justify trial design, or even serve as primary evidence in select use cases, such as label expansions or niche indications where traditional trials are impractical. From a clinical perspective, they also enable unprecedented levels of personalization, dynamically adapting digital optimization and interventions based on individual patient trajectories. As a result, PDTs and smart medicines developed with in silico intelligence are uniquely positioned to usher in a new era of precision therapeutics, one that is faster, smarter, and fundamentally more patient-centric.

Building trust and infrastructure for the in silico era

Skeptics are right to ask: can we trust these models? The answer lies in validation, transparency, and oversight. In silico tools must be rigorously benchmarked against real-world outcomes, and their algorithms openly scrutinized. Bias in input data can lead to bias in output, and black-box systems must give way to explainable AI. To unlock the full potential of in silico trials, we need standardized frameworks akin to Good Clinical Practice guidelines. Regulatory-grade validation pipelines, shared digital twin libraries, and public-private consortia can ensure consistency, quality, and trust.

The trial of the future will begin digitally. Phase 0 will become “Phase In Silico.” Dose-ranging studies will be performed on virtual cohorts. Only the most promising therapies will advance to confirmatory human trials with smaller sample sizes. It is not just feasible, it is already happening in early pilots. We are living through the dawn of a new paradigm in medicine. In silico research is not just a tool, it is a philosophy. A belief that simulation, modeling, and digital insight can complement and, in many cases, replace the risks and limitations of human experimentation. In a decade, failing to run in silico trials may not just be seen as a missed opportunity. It may be malpractice.

In conclusion, in silico research is not just a tool, it is a paradigm shift, signaling a future where trials begin in simulation and only the most promising therapies move forward to human testing. As regulatory agencies embrace model-informed approaches and computational evidence gains parity with traditional data, the medical community must follow. The ethical, economic, and scientific justifications are clear: simulation is no longer optional, it is essential. To fully realize this transformation, we must invest in robust infrastructure, demand transparency and validation, and equip the next generation of clinicians and scientists to lead in the digital era. The end of human trials is not the end of science, but the beginning of a smarter, safer, and more personalized future for medicine.
